# Retraction Note to: Inter- and intraobserver agreement of the quantitative assessment of [^99m^Tc]-labelled anti-programmed death-ligand 1 (PD-L1) SPECT/CT in non-small cell lung cancer

**DOI:** 10.1186/s13550-021-00851-1

**Published:** 2021-10-12

**Authors:** Daniel Johnathan Hughes, Gitasha Chand, Vicky Goh, Gary J. R. Cook

**Affiliations:** 1 Department of Cancer Imaging, School of Biomedical Engineering and Imaging Sciences, King’s College London, 4th Floor, Lambeth Wing, St Thomas’ Hospital, Westminster Bridge Road, London, SE1 7EH UK; 2 King’s College London & Guy’s and St. Thomas’ PET Centre, London, UK; 3 Department of Research and Development, NanoMab Technology Limited, Shanghai, People’s Republic of China; 4 Department of Nuclear Medicine, Shanghai General Hospital, Shanghai Jiaotong University School of Medicine, Shanghai, People’s Republic of China; 5 Department of Radiology, Guy’s and St. Thomas’ NHS Foundation Trust, London, UK

## Retraction Note: EJNMMI Res (2020) 10:145 10.1186/s13550-020-00734-x

The authors have retracted this article [1] as they did not have permission to use and publish the data. All authors agree to this retraction.
